# Rescue of Mitochondrial Function in Hutchinson-Gilford Progeria Syndrome by the Pharmacological Modulation of Exportin CRM1

**DOI:** 10.3390/cells12020275

**Published:** 2023-01-10

**Authors:** Feliciano Monterrubio-Ledezma, Fernando Navarro-García, Lourdes Massieu, Ricardo Mondragón-Flores, Luz Adriana Soto-Ponce, Jonathan J. Magaña, Bulmaro Cisneros

**Affiliations:** 1 Department of Genetics and Molecular Biology, Centro de Investigación y de Estudios Avanzados del Instituto Politécnico Nacional (CINVESTAV), Mexico City 07360, Mexico; 2 Department of Cell Biology, Centro de Investigación y de Estudios Avanzados del Instituto Politécnico Nacional (CINVESTAV), Mexico City 07360, Mexico; 3 Department of Neuropathology, Instituto de Fisiología Celular, Universidad Nacional Autónoma de México (UNAM), Mexico City 04510, Mexico; 4 Department of Biochemistry, Centro de Investigación y de Estudios Avanzados del Instituto Politécnico Nacional (CINVESTAV), Mexico City 07360, Mexico; 5 Departament of Bioengineering, Escuela de Ingeniería y Ciencias, Tecnológico de Monterrey-Campus Ciudad de México, Ciudad de Mexico 14380, Mexico; 6 Laboratory of Genomic Medicine, Department of Genetics, National Rehabilitation Institute-Luis Guillermo Ibarra Ibarra (INR-LGII), Mexico City 14389, Mexico

**Keywords:** Hutchinson-Gilford progeria syndrome, mitochondria, CRM1, leptomycin B, mitophagy, PGC-1α, lysosomes, LAMP2

## Abstract

Hutchinson-Gilford progeria syndrome (HGPS) is a rare premature aging disorder caused by the expression of progerin, a mutant variant of Lamin A. Recently, HGPS studies have gained relevance because unraveling its underlying mechanism would help to understand physiological aging. We previously reported that the CRM1-mediated nuclear protein export pathway is exacerbated in HGPS cells, provoking the mislocalization of numerous protein targets of CRM1. We showed that normalization of this mechanism by pharmacologically inhibiting CRM1 with LMB (specific CRM1 inhibitor), mitigates the senescent phenotype of HGPS cells. Since mitochondrial dysfunction is a hallmark of HGPS, in this study we analyze the effect of LMB on mitochondrial function. Remarkably, LMB treatment induced the recovery of mitochondrial function in HGPS cells, as shown by the improvement in mitochondrial morphology, mitochondrial membrane potential, and ATP levels, which consequently impeded the accumulation of ROS but not mitochondrial superoxide. We provide evidence that the beneficial effect of LMB is mechanistically based on a combinatory effect on mitochondrial biogenesis via upregulation of PGC-1α expression (master transcription cofactor of mitochondrial genes), and mitophagy through the recovery of lysosomal content. The use of exportin CRM1 inhibitors constitutes a promising strategy to treat HGPS and other diseases characterized by mitochondrial impairment.

## 1. Introduction

The function of eukaryotic cells largely depends on the physical and functional separation between the nucleus and the cytoplasm by the nuclear envelope (NE). The NE comprises the outer nuclear membrane, the inner nuclear membrane (INM), the nuclear pore complex (NPC) and the nuclear lamina, which is a filamentous network underlying the INM that is constituted by lamin types A and B [1]. Communication between the nuclear and cytoplasmic compartments takes places through the bidirectional trafficking of molecules across the NPC. In addition to the major structural role played in the nucleus, lamins are involved in key processes, including genome organization, DNA replication, gene expression and cell cycle regulation [2]. The critical role of lamins in cell/tissue function is highlighted by the identification of a variety of human diseases caused by mutations on their encoding genes, so called laminopathies, including muscular dystrophy, lipodystrophy, and premature aging syndromes [3].

Hutchinson-Gilford progeria syndrome (HGPS) is a rare premature aging disorder that recapitulates the distinctive features of physiological aging, including bone (osteopenia and osteoporosis), muscle (sarcopenia), skin (lipodystrophy), metabolic (high energy expenditure), and cardiovascular (atherosclerosis) alterations [4,5]. There is no cure for HGPS so far, and affected children die at around 13 years of age due to myocardial attack or stroke [6,7]. The genetic basis of HGPS is a spontaneous single-nucleotide substitution (1824 C > T) within the *LMNA* gene, located at chromosome 1q21.2-q21.3

The mutation activates a cryptic donor splicing site within exon 11, which drives the synthesis of a prelamin A mRNA containing an internal deletion of 150 base pairs. This alternative transcript leads, in turn, to the translation of a mutant variant of lamin A termed progerin, which lacks 50 amino acids at the C-terminus. The maturation of pre-lamin A includes farnesylation, proteolysis and carboxymethylation on its C-terminal CaaX motif, and finally the cleavage of the modified C-terminus by the zinc metalloprotease ZMPSTE24. Progerin instead is permanently farnesylated, because its truncated C-terminus lacks the ZMPSTE24 cleavage site of lamin A. Farnesylation confers on progerin a dominant negative function, and it aberrantly anchors to the NE, thereby disturbing a plethora of cellular processes, including genome stability, chromatin organization, cell cycle regulation and gene expression [3,8].

We previously unveiled an impairment in the nuclear protein export pathway in primary fibroblasts from HGPS patients [9]; HGPS cells exhibited an enhanced nuclear protein export activity due to the progerin-driven overexpression of exportin-1 (XPO1), also known as chromosomal region maintenance 1 (CRM1). CRM1 is the major transport receptor that exports proteins carrying a hydrophobic-rich nuclear export signal (NES) from the nucleus to the cytoplasm through the NPC [10,11]. The hyperactivity of CRM1 gives rise to an imbalance in the partition of critical proteins (transcription factors and structural proteins) between the cytoplasm and nucleus [12]. Remarkably, we showed that pharmacological inhibition of CRM1, using the specific CRM1 inhibitor Leptomycin B (LMB) [11], resulted in the alleviation of several senescent marks in HGPS cells, including aberrant nuclear morphology, nucleolar expansion, loss of perinuclear chromatin, Lamin B1 downregulation, and senescent cellular morphology [9].

Owing to the central role of mitochondria in cell physiology, acting as a hub for energy generation, reactive oxygen species (ROS) homeostasis and senescence [13], evaluation of LMB as a potential therapeutic agent against HGPS must include the analysis of mitochondrial function. Furthermore, gathering evidence suggests a causative association between mitochondrial dysfunction and pathological aging; for instance, the study of a mtDNA mutator mouse model showed that an increased level of mtDNA mutations contributes to progeroid phenotypes [14,15]. In fact, a severe dysfunction of mitochondria has been described in HGPS cells, which results at least in part from the progerin-mediated accumulation of ROS [16,17,18,19]. The altered mitochondrial function of HGPS cells is characterized by the aberrant morphology and low mobility of mitochondria, which is accompanied with low ATP levels [16,17,18,19]. Furthermore, a decrease in mitogenesis due to low expression of the central transcriptional regulator of mitochondrial homeostasis, peroxisome proliferator-activated receptor-gamma coactivator-1 alpha (PGC-1α), was found in HGPS fibroblasts [20]. Therefore, the aim of this study was to evaluate the impact of the LMB-mediated pharmacological modulation of CRM1 on mitochondrial function in HGPS fibroblasts.

## 2. Materials and Methods

### 2.1. Cell Culturing and Drug Treatment

Normal (AG08469) and HGPS (AG11513 and AG11498) human dermal fibroblasts were acquired from Coriell Cell Repositories (Camden NJ). Untransformed HGPS cell cultures carry the classic G608G splice site mutation at the LMNA gene; AG11513 was obtained from a leg skin biopsy of an 8-year-old White American, while AG11498 was obtained from a thigh skin biopsy of a 14-year-old Black/African American. Primary fibroblasts were cultured in a humidified 5% CO_2_ atmosphere in Minimal Essential Medium Eagle (MEM) (Invitrogen, Carlsbad, CA, USA) supplemented with 15% fetal bovine serum (Invitrogen, USA), pyruvate 1 mM (Sigma, Irvine, UK), 10 U/mL penicillin and 10 µg/mL streptomycin (Sigma, Saint Louis, MO, USA). When indicated, fibroblast cultures were treated for three days with 1 nM leptomycin B (LMB; Sigma-Aldrich, MO, USA) diluted in Et-OH. This concentration of LMB was previously standardized based on both its beneficial effect on the senescent phenotype of HGPS fibroblasts, and its low toxicity [9]. Fresh medium was added twice a week and cultures at 95% confluency were passaged at a 1:3 ratio.

### 2.2. Antibodies

The following primary antibodies were used in this study: rabbit polyclonal antibodies directed against PGC1-α (Thermo Fisher Scientific, Waltham, MA, USA, [cat: PA5-72948]), LC3 (MBL International, Woburn, MA, USA, [cat: PD014]) and LAMP2 (Millipore, Sigma-Aldrich, St Louis, MO, USA, [cat: L0668]). Mouse monoclonal antibodies directed against TOM20 (Santa Cruz Biotechnology, CA, USA, [cat: sc-17764]) included PINK1 (Santa Cruz Biotechnology, CA, USA, [cat: sc-38CT20,8.5]), Parkin (Santa Cruz Biotechnology, CA, USA, [cat: sc-32282]), TFEB (Santa Cruz Biotechnology, CA, USA, [cat: sc-166736]) and actin (a gift from Dr. Manuel Hernández, CINVESTAV-IPN, Mexico city Mexico).

### 2.3. Western Blotting

Fibroblast lysates were electrophoresed on 12% or 15% (~15 kDa proteins) SDS-polyacrylamide gels and transferred onto nitrocellulose membranes (Cat: 1620115, Bio-Rad, Hercules, CA, USA). The membranes were blocked in TBST [100 mM Tris-HCl pH 8.0, 150 mM NaCl, 0.5% (*v*/*v*) Tween-20] with 5% low-fat dried milk and incubated overnight at 4 °C with the proper primary antibodies. The specific protein signal was developed using the corresponding secondary antibodies and the enhanced chemiluminescence (ECL™) Western blotting detection system (Amersham Pharmacia, GE Healthcare), according to the manufacturer’s instructions.

### 2.4. Indirect Immunofluorescence and Confocal Microscopy Analysis

Cells grown on coverslips at 70% confluence were fixed with 4% paraformaldehyde (PFA) for 10 min, permeabilized with 0.2% Triton X-100 in PBS for 10 min and blocked with 5% BSA in PBS for 20 min at room temperature. After that, cells were incubated overnight at 4 °C with the proper primary antibodies and then with the corresponding secondary fluorochrome-conjugated antibodies for 2 h at room temperature (Jackson Immunoresearch Laboratories, West Grove, PA, USA). For colocalization of mitochondria with LC3, cells seeded on coverslips at 70% of confluence were stained with MitoTracker Red CMXRos for 15 min at 37 °C in fresh medium under humidified atmosphere with 5% CO_2_, prior to proceeding with fixation for 10 min with 4% PFA. Cells were then washed twice with PBS and further subjected to immunostaining with anti-LC3 antibodies, as mentioned above. To stain nuclei, cells were incubated for 10 min at room temperature with 1 mg/mL Diamidino-2-phenylindole (DAPI) (Sigma-Aldrich, St. Louis, MO, USA) in PBS. After washing, coverslips were mounted on microscope slides with VectaShield (Vector Laboratories, Inc., Burlingame, CA, USA) and visualized in an epi-fluorescence microscope. Images were acquired using an Eclipse Ti-E inverted confocal laser scanning microscope (NiKon, Tokyo, Japan).

### 2.5. Transmission Electron Microscopy

Briefly, the cells previously washed with PBS (138 mM NaCl, 1.1 mM K_2_PO_4_, 0.1 mM Na_2_HPO_4_, and 2.7 mM KCl, at pH 7.2), were fixed with 2.5% glutaraldehyde in PBS for 1 h at room temperature and incubated for 1 h in 1% OsO_4_ at 4 °C, suspended in PBS and then thoroughly washed with PBS. Samples were gradually dehydrated in increasing concentrations of ethanol, embedded in Spurr’s resin (Electron Microscopy Sciences, Washington, DC, USA), and polymerized at 60 °C for 48 h. Thin sections were obtained using a Reichert Jung ultramicrotome (Reichert Jung, Vienna, Austria) on 100 mesh copper grids and stained with uranyl acetate and lead citrate. The samples were analyzed and examined in the transmission electron microscope JEM 1400 at 80keV (JEOL. Ltd., Tokyo, Japan). The evaluation of electron microscopy images was carried out by double-blind visual observation.

### 2.6. Live Imaging of Mitochondria and Lysosomes

To evaluate mitochondrial morphology, lysosomal content and mitochondria-lysosome colocalization, fibroblasts plated on 35-mm glass-bottom dishes at 70% confluence were synchronized at the G0/G1 phase by culturing them in MEM medium supplemented with a low percentage of serum (0.1% medium) for 12 h. Afterwards, cells were washed with PBS twice and cultured for 12 h in fresh medium with the normal percentage of serum (15%). Cells were further stained for 25 min with 100 nM MitoTracker Green (Thermo Fisher; Cat: M7514, Waltham, MA, USA) or 50 nM Lysotracker DND-99 (Thermo Fisher; L7528, USA) or with both MitoTracker Green and Lysotracker. Cells were then washed twice with PBS and further stained for 10 min with Hoechst (Sigma Aldrich; Cat: B2261, St. Louis, MO, USA) prepared at 1 µg/mL in fresh medium. Cell preparations were washed twice with PBS again and maintained in fresh medium at 37 °C under a humidified atmosphere with 5% CO_2_ during imaging. Live imaging acquisition was conducted using the Eclipse Ti-E inverted confocal laser scanning microscope (NiKon, Japan), equipped with a Tokai Hit INU on-stage incubator (Spectra Services, Ontario, NY, USA) and C90 camera. Morphometric analysis of mitochondria and lysosomal counting was performed using Image J 1.46j software with a plugin to count, determine area, circularity and Feret’s diameter (which is the longest distance between any two points along the selection boundary) parameters, as previously reported [21,22]. These allowed for the setting of the parameters of the ‘analyze particles’ plug-in as follows: size (area) from 0.1 to 20 μm^2^ and circularity from 0.1 to 1.0.

### 2.7. Determination of the Mitochondrial Membrane Potential (ΔΨ_m_)

To assess ΔΨ_m_, fibroblasts plated on 35-mm glass-bottom dishes were treated with LMB or the vehicle alone for 3 days. Cells were then stained with 5,5,6,6′-tetrachloro-1,1′,3,3′ tetraethylbenzimidazoyl-carbocyanine iodide (JC-1) 500 nM (Invitrogen; Cat: T3168, USA) for 30 min at 37 °C in a 5% CO_2_ atmosphere. JC-1 dye accumulates in mitochondria in a concentration-dependent fashion. Cells were subjected to live imaging using confocal microscopy (LSM 800, ZEISS; Lausanne, Switzerland), and fluorescence emission was measured at 529 nm (green fluorescence generated by the monomeric form of JC-1) and at 590 nm (red fluorescence generated by JC-1 aggregates). Mitochondrial depolarization was calculated by the 590 nm/529 nm fluorescence intensity ratio using Image J.

### 2.8. Intracellular Determination of Reactive Oxygen Species (ROS)

Intracellular levels of ROS were detected using the Cellular ROS Assay Kit (Abcam; Cat: ab113851, Cambridge, UK), according to the manufacturer’s instructions. In brief, fibroblasts were plated on 35-mm glass-bottom dishes at 70% confluence; they were stained with 40 µM 2’-7’-dichlorodihydrofluorescein-diacetate (DCFH-DA, diluted in culture medium), in the dark for 45 min at 37 °C with 5% CO_2_. Cells were counterstained for 15 min with Hoechst to visualize the nuclei as mentioned previously, washed twice with PBS, and subjected to imaging using a confocal microscope. Green-stained live cells correspond to cells positive for reactive oxidative species. The intracellular fluorescence intensity was measured using Image J software, and data were plotted using GraphPad Prism 8.

### 2.9. Determination of Mitochondrial ROS

To assess mitochondrial superoxide, fibroblasts were stained with MitoSox (M36008; (Thermo Fisher Scientific, MA, USA), as previously described [23]. Briefly, fibroblasts were plated on 60-mm dishes that were treated with LMB or the vehicle alone for 3 days. Cells were trypsinized and a concentration of 500,000 cells per ml was placed into 1.5 mL flow cytometry tubes for staining with 1 µM MitoSox for 20 min at 37 °C. Stained cells were then subjected to flow cytometry analysis in an Attune Acoustic Focusing Cytometer (Applied Biosystems, Life Technology; Carlsbad, CA, USA). The emission of red fluorescence is indicative of mitochondrial superoxide production.

### 2.10. Quantification of ATP

Intracellular ATP was measured using a luminescent ATP detection assay kit (Abcam, ab113849) according to the manufacturer’s instructions. Briefly, cells plated onto 96-well plates (1 × 10^5^ cells/well) were lysed and exposed to a substrate solution for measuring luminescence intensity in a Fluoroskan Scent FL Microplate Luminometer (Thermo Fisher Scientific, MA, USA). ATP content was proportional to the luminescence counts.

### 2.11. RNA Extraction and Real-Time Quantitative PCR (qRT-PCR)

Total RNA was obtained from WT and HGPS cells using the Direct-zol RNA MiniPrep kit (Zymo Research, Irvine, CA, USA), according to the manufacturer’s instructions. The RNA yield and purity were assessed in a NanoDrop ND-1000 spectrophotometer (Thermo Fisher Scientific, Inc.). The qRT-PCR assays were carried out on the Step One Plus Real Time PCR System (Applied Biosystems, Foster, CA, MA), using the KAPA SYBR Fast One Step qRT-PCR kit (Kapa Biosystems; Wilmington, MA, USA) and a standard protocol for PCR cycling. The PGC-1α expression levels were quantified using the comparative 2^ΔΔct^ method and β-actin as an endogenous control. Primer sequences were as follows: PGC-1α, forward 5′-ATCCTCTTCAAGATCCTGCT-3′, reverse 5′-GACTCTCGCTTCTCATACTCTC-3′; actin; forward 5′-CAGACAGCGAAAGGATGAG-3′, reverse 5′-CAGACAGCGAAAGGATGAG-3′.

## 3. Results

### 3.1. Restoration of Mitochondrial Morphology in HGPS Cells by the LMB-Mediated Pharmacological Inhibition of Exportin CRM1

**Figure 1:**
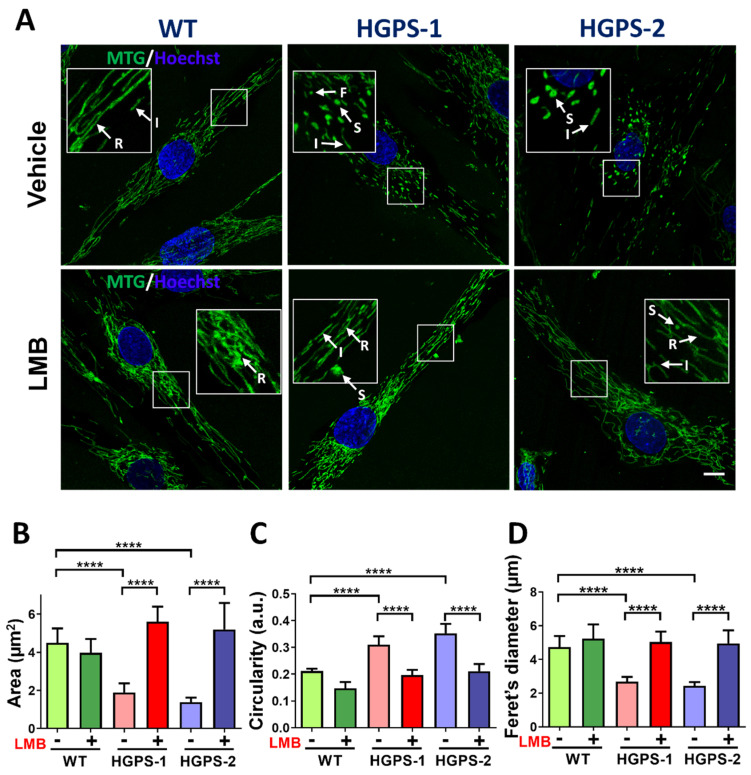
Treatment with LMB recovers mitochondrial morphology in HGPS cells. (**A**) WT, HGPS-1, and HGPS-2 primary fibroblasts grown on coverslips were treated for 3 days with 1 nM LMB or the vehicle alone, and then subjected to MTG staining. Nuclei were labeled with Hoechst prior to confocal microscopy analysis. Typical fluorescence images of MTG-stained mitochondria are shown. Arrows indicate; F: fragmented; I: intermediate; R: reticulate; and S: swollen mitochondria. Scale bar, 10 µm; insets, 4×. (**B**–**D**) Morphometric analysis of mitochondria from WT and HGPS 1-2 cells was conducted to calculate area, circularity, and Ferret’s diameter parameters. Results correspond to the mean +/− SD from three separate experiments (n = 80 cells per condition) with significant differences determined by unpaired *t* test (n = 50 cells per experimental group; **** *p* < 0.0001).

To ascertain whether the LMB-driven pharmacological modulation of CRM1 improves mitochondrial morphology in HGPS cells, WT, HGPS-1 and HGPS-2 fibroblasts were treated with LMB or the vehicle alone for 3 days. Cells were synchronized at the G0/G1 phase because mitochondrial morphology changes during cell cycle progression [23]. Afterwards, cells were incubated with MitoTracker Green dye (MTG), which stains mitochondria regardless of the mitochondrial membrane potential (ΔΨ_m_) in living cells, to evaluate their morphology. Most WT fibroblasts exhibited mitochondrial reticular networks with long tubules, while HGPS fibroblasts contained numerous short intermediate mitochondrial tubules and fragmented and swollen mitochondria, which likely correspond to damaged mitochondria (Figure 1A).

**Figure 2:**
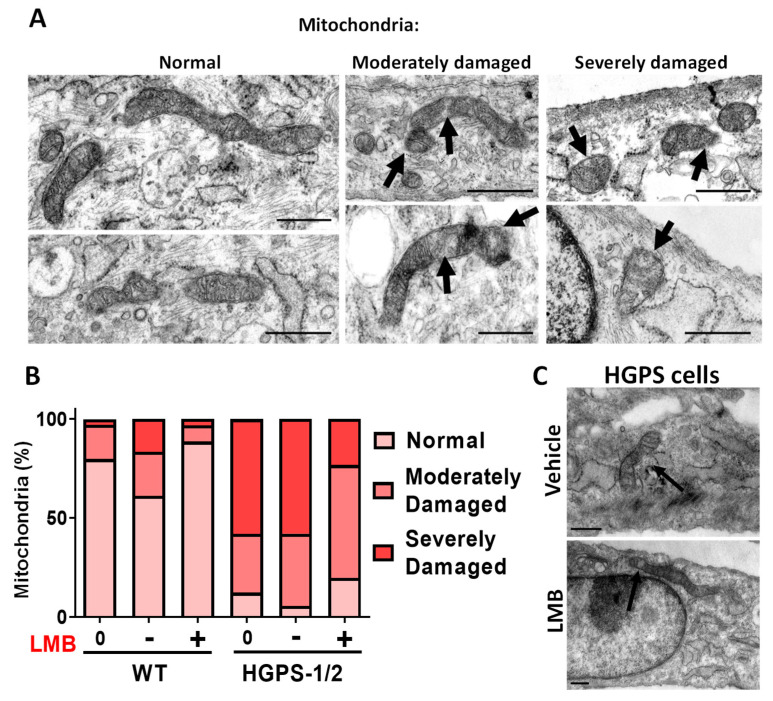
Electronic microscopy (EM) analysis of mitochondria in WT and HGPS cells subjected to LMB treatment. Primary fibroblast cell cultures were incubated for 3 days with LMB or the vehicle alone as in Figure 1, before EM imaging. (**A**) Typical transmission electron micrographs are shown (scale bar, 500 nm). Mitochondria were evaluated for the integrity of the membrane, matrix and cristae, and were classified as mitochondria with normal morphology; moderately damaged mitochondria (moderate alterations in the membrane, and the presence of inclusions in the matrix); and severely damaged mitochondria (severe alterations in the membrane shape with increased mitochondrial volume [swollen], and alteration of the cristae with internal aggregates). Arrows point to the aberrant mitochondrial structures. (**B**) The percentage of mitochondria with each morphological pattern found in WT and HGPS 1–2 cell cultures treated with LMB (+) or the vehicle alone (−), as well as untreated cells (0), is shown (n > 100 mitochondria per experimental condition). (**C**) Typical EM images of HGPS cells treated with LMB or the vehicle alone are shown (scale bar, 500 nm). Arrows point to the aberrant (vehicle-treated HGPS cells) or restored (LMB-treated HGPS cells) mitochondrial structures.

Interestingly, most HGPS fibroblasts upon treatment with LMB showed mitochondrial elongated tubules and reticular networks, which closely resembled WT mitochondrial morphology. The quantification of mitochondrial morphology using different morphological parameters confirmed that treatment with LMB prevented morphological aberrations of mitochondria: an increase in both mitochondrial area and Ferret’s diameter and a decrease in mitochondrial circularity were found in LMB-treated HGPS cells, compared with vehicle-treated HGPS cells (Figure 1B–D). The LMB-treated HGPS cell parameters were comparable to WT cells. Furthermore, the analysis of mitochondrial morphology at the structural level was conducted under electron microscopy observation. WT cells mainly contained normal mitochondria (about 80%) with typical double membrane and intact matrix with visible cristae; occasionally, normal cells showed moderately damaged mitochondria characterized by alterations in the integrity of the mitochondrial membrane, inclusions in the matrix and alterations in some cristae (17%), and severely damaged mitochondria (3%), which included swollen mitochondria with loss of the inner membrane cristae and the presence of internal aggregates (Figure 2A,B). Ethanol used as vehicle for LMB caused a change in these ratios of normal (61%), moderately (22%), and severely (17%) damaged mitochondria, possibly due to its uncharacterized effect on mitochondria [24].

Remarkably, the use of LMB on WT cells clearly improved the mitochondrial morphology ratios as compared to vehicle-treated cells: normal (88%), moderate (8%), and severely (3%) damaged mitochondria. On the other hand, most HGPS 1–2 cells had both moderate damaged mitochondria, defined by tubular morphology with a disrupted double membrane and the presence of inclusions in the matrix, and severe damaged mitochondria, characterized by fragmented and swollen mitochondria with internal aggregates and total disruption of the cristae membrane (Figure 2A,B). Remarkably, HGPS 1–2 cells after LMB treatment showed a significant decrease in the number of severely damaged mitochondria (from 58% to 23%) with the corresponding increase of mitochondria with normal (from 5% to 19%) or moderate-damaged morphology (from 36% to 56%) (Figure 2B).

### 3.2. LMB-Mediated Pharmacological Inhibition of Exportin CRM1 Evokes Recovery of Mitochondrial Function in HGPS Fibroblasts

**Figure 3:**
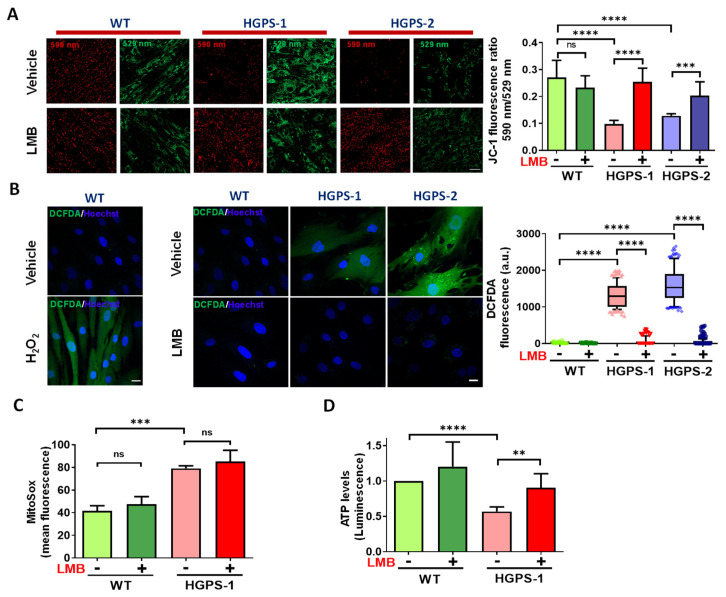
Mitochondrial function is restored in LMB-treated HGPS fibroblasts. (**A**) Δ_Ψm_ of WT and HGPS cells was analyzed using JC-1 dye staining and confocal microscopy analysis. ***Left**.* WT and HGPS1-2 cells previously treated with 1 nM LMB (+) or the vehicle alone (−) for 3 days were stained with JC-1. Typical images of JC-1-stained cells are shown; scale bar, 50 µm. ***Right***, ΔΨ_m_ was calculated by the 590 nm/529 nm fluorescence intensity ratio. Data corresponds to the mean +/− SD of three independent experiments (n≈120 cells per condition), with *p* values denoting significant differences (unpaired *t* test; *** *p* < 0.001; **** *p* < 0.0001). (**B**) ***Left***. The generation of intracellular ROS was measured in WT cells incubated with H_2_O_2_ or its vehicle alone using the H2DCFDA-based assay and confocal microscopy analysis. Nuclei were stained with Hoechst. *Middle*. WT and HGPS1-2 cells were treated with LMB (+) or the vehicle alone (−), as in panel A, prior to being incubated with H2DCFDA to detect ROS. Nuclei were visualized by staining with Hoechst and typical images of H2DCFDA-stained cells are shown. Scale bar, 10 µm. ***Right***. Variation in H2DCFDA fluorescence intensity between the experimental groups was determined using Image J (see Methods for details). Data from three independent experiments is shown in box and whiskers graph (percentile: 10–90, n = 130 cells per condition), and significant differences were obtained using a nonparametric Mann–Whitney test; **** *p* < 0.0001). (**C**) Mitochondrial superoxide production was assessed using MitoSox staining followed by flow cytometry analysis. WT and HGPS-1 fibroblast cultures were treated with 1 nM LMB (+) or the vehicle alone (−) for 3 days prior to staining with 1 µM MitoSox, and the mean value of fluorescence intensity was plotted. Data corresponds to the mean +/− SD of three independent experiments (10,000 events per run), with *p* values denoting significant differences (unpaired *t* test; *** *p* < 0.001). (**D**) The levels of ATP were assessed in WT and HGPS-1 cells previously treated with LMB (+) or the vehicle alone (−) as per panel A. Data correspond to the mean +/− SD of luminescent intensity from three separate experiments, with *p* values denoting significant differences (unpaired *t* test; ** *p* < 0.01; **** *p* < 0.0001); ns, no significance.

Mitochondrial morphology is closely related to their bioenergetics activity [25]. We were then prompted to ascertain whether LMB-mediated restoration of mitochondrial morphology after LMB treatment is reflected in an improvement in their function by measuring different mitochondrial parameters. The mitochondrial membrane potential (ΔΨ_m_) was evaluated by staining cells with JC-1, a fluorescent dye that accumulates in the mitochondria of living cells in a concentration-dependent manner. Functional mitochondria form JC-1 aggregates that display excitation and emission in the red spectrum (590 nm), while defective/depolarized mitochondria are unable to form JC-1 aggregates and JC-1 dye monomers show excitation and emission in the green spectrum (529 nm). Thus, the red/green fluorescence intensity ratio of JC-1 is a suitable measure for the mitochondrial polarization state [26]. As a positive control, WT cells were incubated with carbonyl cyanide 4-(trifluoromethoxy)phenylhydrazone (FCCP), an uncoupling agent for oxidative phosphorylation. As expected, a low 590 nm/529 nm fluorescence ratio was found upon the exposure of WT cells to FCCP, which implies the loss of ΔΨ_m_ (Supplementary Figure S1A). On the other hand, HGPS-1 and HGPS-2 cells showed a drastic decrease of the JC-1 590 nm/529 nm fluorescence ratio, compared with WT cells, indicating the presence of mitochondria with disrupted ΔΨ_m_ (Figure 3A). Interestingly, treatment with LMB significantly restored the ΔΨ_m_ of HGPS-1 and HGPS-2, as shown by their increased 590 nm/529 nm fluorescence ratio, compared with vehicle-treated HGPS cells (Figure 3A). WT cells instead showed no changes in ΔΨ_m_ in response to LMB. As oxidative stress produced by reactive oxygen species (ROS) is a determinant of ΔΨ_m_ depolarization [27], we next analyzed whether the beneficial effect of LMB on HGPS cells is ultimately reflected in ROS levels. The generation of intracellular ROS was visualized by confocal microscopy using the cell permeable ROS-sensitive fluorescence dye H2DCFDA. As a positive control, WT cells were incubated with hydrogen peroxide (H_2_O_2_) to test H2DCFDA sensitivity to detect intracellular ROS. High intensity of H2DCFDA fluorescence was observed inside the H_2_O_2_-treated WT cells, while untreated WT cells showed no perceptible fluorescence (Figure 3B, left panel), which demonstrated the feasibility of H2DCFDA to detect ROS. Significantly higher levels of ROS-dependent H2DCFDA fluorescence was found in HGPS-1 and HGPS-2 cells, compared with WT cells (Figure 3B, middle panels); remarkably, the intensity of H2DCFDA fluorescence virtually disappeared in LMB-treated HGPS-1 and HGPS-2 cells (Figure 3B, middle panels). A quantitative analysis showed that H2DCFDA fluorescence was clearly absent in LMB-treated HGPS cells as observed in the WT cells (Figure 3, right panel). Since H2DCFHDA mainly detect the NADPH-oxidases-mediated cytoplasmic ROS (ROS derived from peroxisomes, endoplasmic reticulum, cell membrane ROS and phagocytes) [28,29], these results imply that treatment with LMB prevented their accumulation in HGPS cells. Next, we ascertained whether LMB has an effect on the ROS generated in mitochondria using MitoSox staining and further quantification by flow cytometry. WT and HGPS-1 cells were stained with two different MitoSox concentrations, so we choose the 1 µM concentration in order to avoid side effects [30] and high noise (Supplementary Figure S1B). As previously reported [16,20], increased mitochondrial superoxide production was observed in HGPS cells; nonetheless, the level of mitochondrial ROS remained unchanged after treatment with LMB in both WT and HGPS-1 cell cultures (Figure 3C). Finally, the synthesis of ATP was evaluated in HGPS-1 cells using a luminescent ATP detection assay. The significant decrease in ATP levels showed by HGPS-1 cells was prevented by LMB treatment (Figure 3D). Overall, these findings imply that the impairment of mitochondrial function exhibited by HGPS cells is rescued via the pharmacological modulation of CRM1.

### 3.3. Pharmacological Modulation of CRM1 Activity Promotes Mitochondrial Biogenesis via Upregulation of PGC-1α Expression in HGPS Fibroblasts

**Figure 4:**
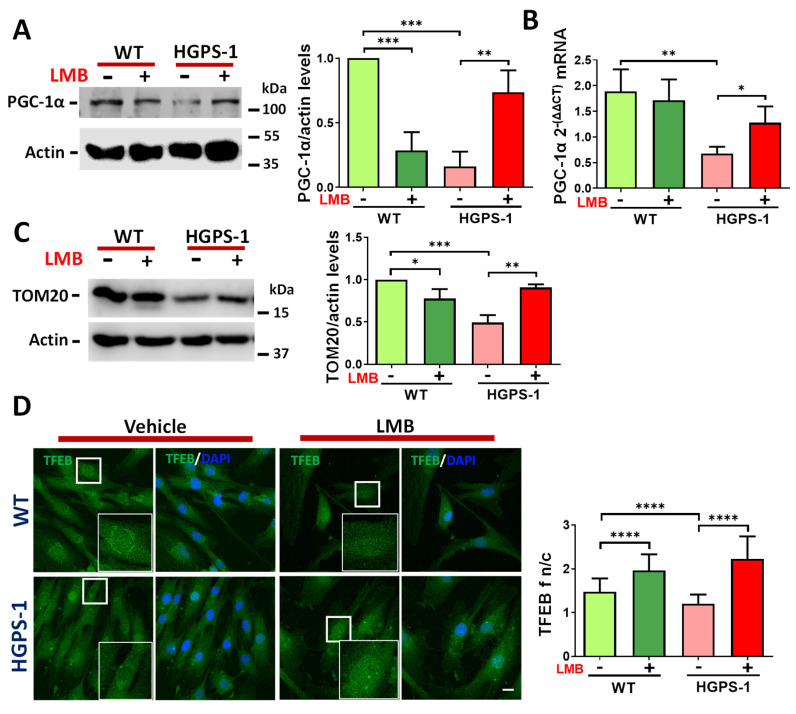
LMB treatment induces PGC-1α expression in HGPS-1 fibroblasts. (**A**) Lysates from WT and HGPS-1 cell cultures treated with LMB or the vehicle alone for 3 days were fractionated by SDS-PAGE and further analyzed by Western blotting using specific antibodies against PGC-1α and actin (loading control). ***Right**.* Relative protein expression of PGC-1α was calculated, and data correspond to the mean +/− SD from three independent experiments (unpaired *t*-test; ** *p* < 0.01; *** *p* < 0.001). The mean value obtained in vehicle-treated WT cells was set at 1, and the mean value of LMB-treated HGPS-1 was plotted with respect to this value. (**B**) The PGC-1α mRNA expression was assessed by qRT-PCR assays (see Methods for details). Data corresponds to the mean +/− SD of 2^ΔΔct^ expressions from three separate experiments (unpaired *t*-test; ** *p* < 0.01; * *p* < 0.05). (**C**) The TOM20 expression level was assayed as in panel A and the Western blotting was performed using specific antibodies against TOM20 and actin as a loading control. ***Right**.* The relative protein expression of TOM20 was calculated, and data correspond to the mean +/− SD from three independent experiments (unpaired *t*-test; * *p* < 0.05; ** *p* < 0.01; *** *p* < 0.001). The mean value obtained in vehicle-treated WT cells was set at 1, and the mean value of LMB-treated HGPS-1 was plotted with respect to this value. (**D**) WT and HGPS-1 cells grown on coverslips were treated with 1 nM LMB or the vehicle alone for 3 days, prior to confocal microscope analysis using TFEB antibodies. Nuclei were stained with DAPI; scale bar 10 µm. Representative images from three independent experiments are shown. **Right**. The TFEB n/c ratio was calculated from three independent experiments (n = 80 cells per condition), and significant differences were calculated by unpaired *t* test; **** *p* < 0.0001.

To ascertain whether the restoration of mitochondrial function is due to an impact of the treatment with LMB on mitochondrial biogenesis, the expression of PGC-1α, a central transcriptional regulator of mitochondrial biogenesis [31,32,33], was analyzed in WT and HGPS-1 fibroblasts treated with LMB or the vehicle alone for 3 days. The expression of PGC-1α drastically declined in HGPS-1 cells at protein level (84%), as well as the mRNA level (64%), compared with WT cells (Figure 4A,B). However, treatment of HGPS-1 cells with LMB partially restored the content of PGC-1α (from 26% to 73%) as well as that of its respective transcript (from 36% to 71%), compared with vehicle-treated HGPS-1 cells (Figure 4A,B). Mitochondrial biogenesis is accompanied by an increased expression of components of the protein import machinery [34]. Therefore, the role of the outer membrane receptor TOM20 (outer mitochondrial membrane protein) was evaluated. Western blot analyses showed that the TOM20 protein levels were decreased by 51% in HGPS cells as compared to WT cells. Consistent with an enhancement in mitochondrial biogenesis, TOM20 was found to increase (from 49% to 90%) in LMB-treated HGPS-1 cells (Figure 4C), which is also indicative of an augment in mitochondrial mass [35]. The immediate consequence of inhibiting CRM1 with LMB is the nuclear retention of protein clients of CRM1. We then envisaged that enhanced PGC-1α expression could be functionally linked to the LMB-mediated nuclear accumulation of transcription factors that activate its promoter in HGPS cells. To approach this notion, we analyzed the cellular distribution of transcription factor EB (TFEB), because it has been implicated in the positive regulation of PGC-1α promoter and is a client of CRM1 [36,37]. In line with our hypothesis, TFEB was found to be mislocalized from the nucleus to the cytoplasm in HGPS cells, but regained nuclear accumulation after LMB treatment (Figure 4D).

### 3.4. The Basal Activation of Mitophagy Shown by HGPS Cells Was Normalized via the LMB-Mediated Inhibition of CRM1

**Figure 5:**
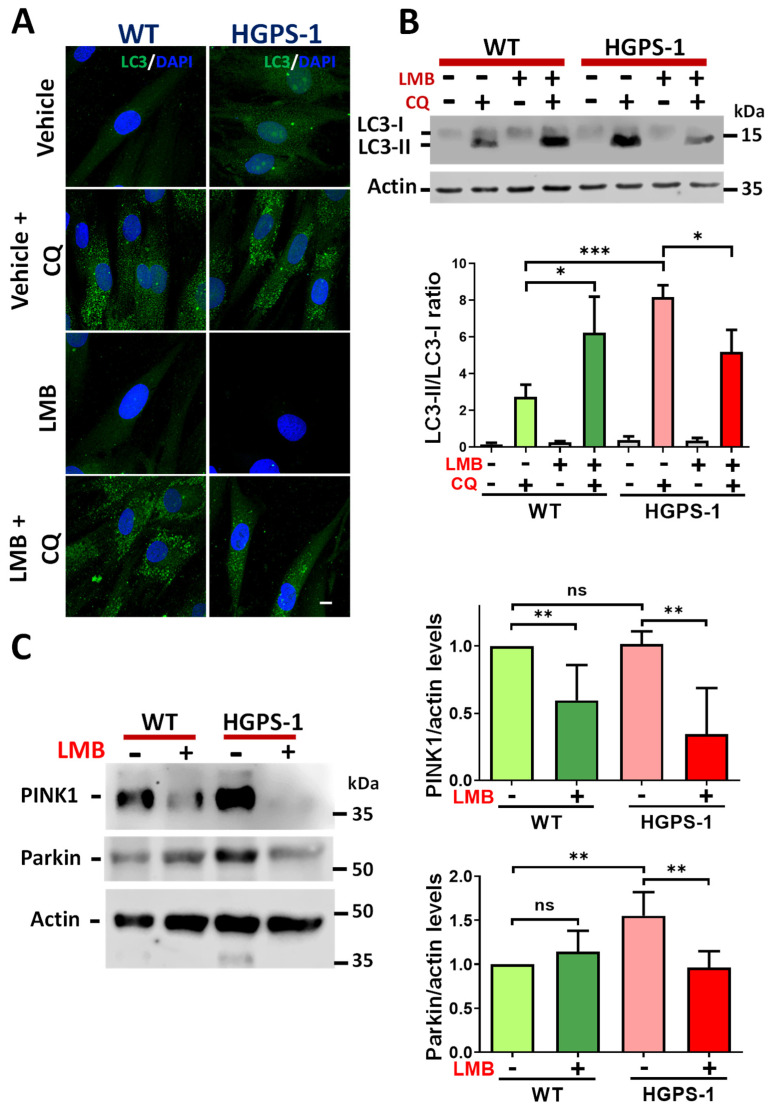
Mitophagy-activation in HGPS cells is mitigated upon treatment with LMB. (**A**) WT and HGPS-1 cells grown on coverslips were treated with 1 nM LMB or the vehicle alone for 3 days or further incubated with 50 µM CQ for 12 h, prior to being subjected to confocal microscope analysis using the LC3 antibody. Representative images from three independent experiments are shown. Nuclei were stained with DAPI; scale bar 10 µm. (**B**) Lysates from WT and HGPS-1 cells treated with LMB and/or with CQ, as in panel A, were analyzed by Western blotting using specific antibodies against LC3 and actin (loading control). ***Bottom***. Quantification of the LC3-II/LC3-I ratio is shown. Data correspond to the mean +/− SD of three independent experiments, with *p* values denoting significant differences (unpaired *t* test; * *p* < 0.05; *** *p* < 0.001). (**C**) PINK1 and parkin levels of WT and HGPS-1 cells, previously treated as in panel A, were analyzed by Western blotting using actin as a loading control. ***Right**.* The levels of PINK1 and parkin were assessed in WT and HGPS-1 cells and graphed. Data represents the mean +/− SD of three separate experiments, with *p* values referring to statistically significant differences (unpaired *t* test; ** *p* < 0.01); ns, no significance.

In addition to mitochondrial biogenesis, the mitochondrial proficiency also depends on mitophagy, a process dedicated to the elimination of damaged mitochondria via autophagy [38,39]. Thus, we determined the condition of mitophagy in HGPS cells, as well as the impact of inhibiting CRM1 activity with LMB on this process. To this end, WT and HGPS-1 cells previously treated with LMB or the vehicle alone were incubated with chloroquine (CQ; inhibitor of the fusion between autophagosomes and lysosomes) to stop autophagic activity and evaluate the accumulation of autophagosomes using the autophagosomal protein marker microtubule-associated protein 1A/1B-light chain 3A (LC3). The cytosolic form of LC3 is called LC3-I, while the phosphatidylethanolamine (PE)-conjugated form of LC3 (LC3-II) is recruited to the membrane of autophagosomes. Thus, the LC3-II/LC3-I ratio accurately monitors the autophagic flux [40]. Increased LC3 puncta and a significant augment of the ratio of LC3-II/LC3-I were found in vehicle-treated HGPS-1 cells by immunofluorescence and Western blot assays, respectively, in comparison with vehicle-treated WT cells (Figure 5A,B). Interestingly, treatment of HGPS-1 fibroblasts with LMB resulted in a decrease of both LC3 puncta and the LC3-II/LC3-I ratio, compared with vehicle-treated HGPS-1 cells (Figure 5A,B). As damaged depolarized-mitochondria are recognized by PTEN-induced kinase 1 (PINK1), which in turn engages parkin to dysfunctional mitochondria to proceed with ubiquitination and further degradation of mitochondrial proteins by lysosomal digestion [38,41], we evaluated the levels of PINK1 and parkin in HGPS-1 cells that were previously treated with LMB or the vehicle alone. Increased levels of parkin but not PINK1 were found in HGPS-1 cells compared with WT cells. Nonetheless, the treatment with LMB clearly reversed the upregulation of parkin (Figure 5C). In addition, LMB caused a decrease of PINK1 level in HGPS-1 and WT cells, compared with both vehicle-treated HGPS-1 and WT cells, respectively.

**Figure 6:**
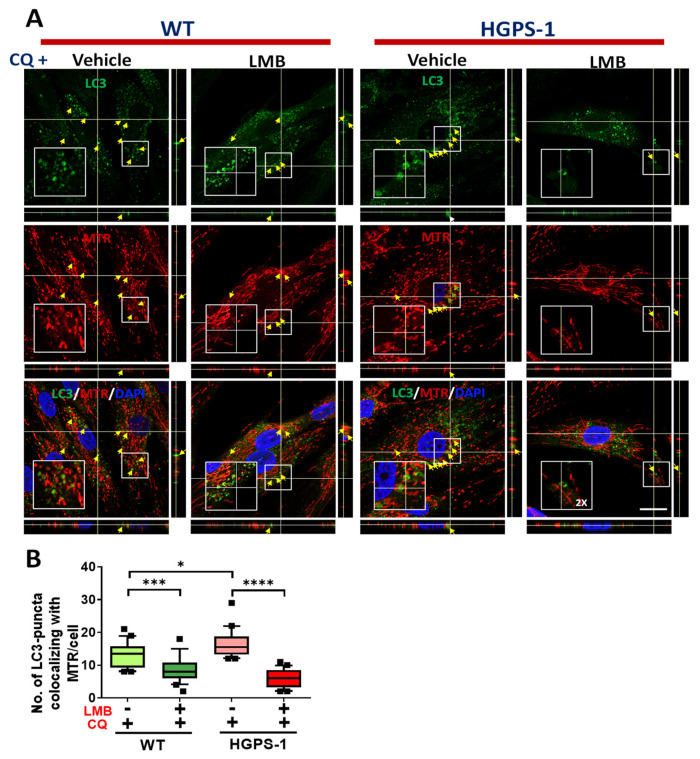
Increased colocalization of LC3 puncta and mitochondria in HGPS-1 cells is normalized in the presence of LMB with an autophagy inhibitor. (**A**) WT and HGPS-1 cells grown on coverslips were treated for 3 days with 1 nM LMB or the vehicle alone, and further incubated with 50 µm CQ for the last 12 h of the treatment, to favor the accumulation of autophagosomes. Cells were then stained with MTR to decorate mitochondria and then fixed and immunolabeled for LC3. Nuclei were stained with DAPI prior to confocal microscopy analysis. Typical orthogonal views of the images from three separate experiments are shown and yellow arrows point at LC3 and mitochondria colocalization. Scale bar 10 µm. (**B**) The number of LC3 puncta colocalizing with mitochondria was quantified and data from three independent experiments is shown in a box and whiskers graph (percentile: 10–90, n = 60 cells per condition). Significant differences were obtained using a nonparametric Mann–Whitney test; * *p* < 0.05; *** *p* < 0.001; **** *p* < 0.0001.

**Figure 7:**
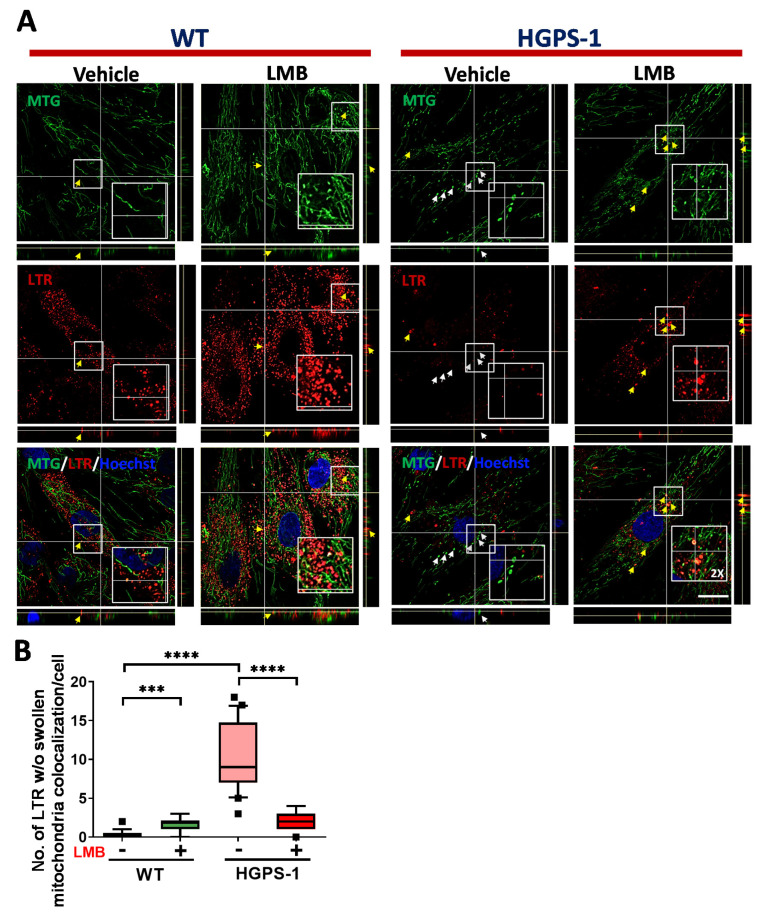
The lack of colocalization of lysosomes with swollen mitochondria in HGPS-1 cells is alleviated by exposure to LMB. (**A**) WT and HGPS-1 fibroblasts were grown on coverslips and incubated with 1 nM LMB or the vehicle alone for 3 days prior to being subjected to cell live analysis. Cells were simultaneously stained with Lysotracker, MTG and Hoechst to visualize the lysosomes, mitochondria, and nuclei, respectively. Typical orthogonal views of the images from three separate experiments are shown. Yellow arrows point at the colocalization between swollen mitochondria and lysosomes, and white arrows point at swollen mitochondria (w/o) colocalization with lysosomes; scale bar 10 µm. (**B**) The number of swollen mitochondria not colocalized with lysosomes was quantified from three independent experiments. Data is shown on a box and whiskers graph (percentile: 10–90, n = 60 cells per condition). The *p* values denoted significant differences (nonparametric Mann–Whitney test; *** *p* < 0.001; **** *p* < 0.0001).

The PINK1/parkin-mediated labeling of depolarized defective-mitochondria is followed by their engulfment by autophagosomes [39]. To assess this, we analyzed the colocalization between mitochondria and LC3 by confocal microscopy, using MitoTracker Red CMXRos (MTR) and LC3 antibodies. The number of cells containing colocalization of mitochondria with LC3 puncta was greater in HGPS-1 cell than WT cells, but significantly lower in LMB-treated HGPS-1 cells than vehicle treated HGPS-1 cells (Figure 6A,B, Supplementary Figure S2). The mitophagy flux is completed by the fusion of autophagosomes carrying defective mitochondria with the lysosome. To analyze this step, the colocalization between mitochondria and lysosomes, labeled with MTG and LysoTracker, respectively, was assessed in WT and HGPS-1 cells treated with LMB or the vehicle alone. LysoTracker is a vital fluorescent dye that specifically accumulates in acidic and intact lysosomes [42]. Clearly, the number of cells containing damaged/swollen mitochondria not colocalizing with lysosomes was higher in HGPS-1 cells than WT cells, but markedly lower in HGPS-1 cells after the treatment with LMB (Figure 7A,B). Collectively, these results are consistent with the idea that mitophagy is basally activated in HGPS-1 cells to remove defective-depolarized mitochondria, and that such activation is no longer needed once mitochondria is restored through LMB treatment.

### 3.5. Altered Abundance of Lysosomes Is Improved by the LMB-Mediated Inhibition of CRM1 in HGPS Cells

**Figure 8:**
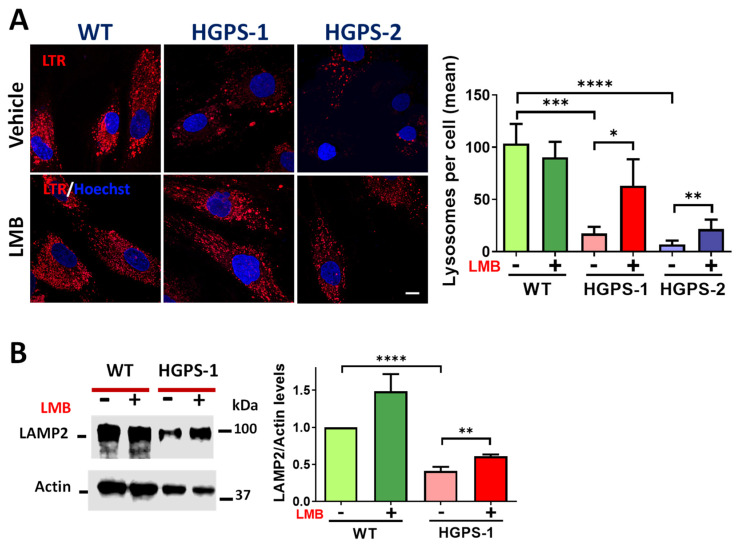
LMB-mediated inhibition of CRM1 improves the lysosomal content of HGPS cells. (**A**) WT and HGPS-1 and HGPS-2 fibroblasts grown on coverslips were treated with LMB or the vehicle alone for 3 days and then stained with Lysotracker and Hoechst to visualize lysosomes and nuclei, respectively. ***Right***. The number of lysosomes per cell was quantified and graphed (n = 50 cells per condition). Data denote significant differences (unpaired *t* test; * *p* < 0.05; ** *p* < 0.01; *** *p* < 0.001; **** *p* < 0.0001). (**B**) Fibroblast lysates were evaluated by Western blotting using specific antibodies for LAMP2 and actin (loading control). ***Right**.* The level of LAMP2 was assessed. Data correspond to the mean +/− SD of three separate experiments, with *p* values denoting significant differences (unpaired *t* test; ** *p* < 0.01; **** *p* < 0.0001).

Since the fusion between autophagosomes and lysosomes is a critical step for mitophagy completion, the lysosome abundance was evaluated in HGPS cells. Lysosome content was examined by cell live analysis using LysoTracker. As shown in Figure 8A, a significant decrease in lysosome number per cell was found in both HGPS-1 and HGPS-2 cells compared with WT cells. Consistently, the level of lysosomal membrane protein 2 (LAMP2) [43] was found to decrease in HGPS-1 cells (Figure 8B). Remarkably, treatment of HGPS-1 with LMB resulted in increased lysosomes per cell and the partial recovery of LAMP2 levels (Figure 8B). Collectively, these results indicate that the LMB-mediated pharmacological inhibition of CRM1 also has an impact on the abundance of lysosomes in HGPS fibroblasts.

## 4. Discussion

Mitochondria are responsible for the generation of cellular energy by producing ATP through oxidative phosphorylation [44]. Mitochondria also regulate multiple cellular processes, including metabolic and reduction-oxidation (redox) homeostasis, cell signaling, apoptosis, and senescence [45]. Highlighting their pivotal role in cell physiology, mitochondrial dysfunction has been involved in a plethora of pathophysiological conditions, including cancer, neurodegenerative diseases, and premature aging syndromes [46]. It is thought that the decline of mitochondrial activity in normal and premature aging is due to the accumulation of mitochondrial DNA mutations, the perturbation of mitochondrial membrane dynamics (fusion and fission) and/or the alteration of the balance between mitochondrial biogenesis and mitophagy [47]. Therefore, mitochondria appear to lose the ability to maintain cellular homeostasis under aging-derived stressful situations [48].

The study of HGPS has provided important evidence of how mitochondrial function deteriorates in pathological aging. HGPS fibroblasts contain fragmented/swollen mitochondria with altered mobility and impaired ΔΨ_m_ [20]. Consistent with their aberrant morphology, the mitochondria of HGPS fibroblasts showed altered respiration [16], reduced ATP levels, the accumulation of ROS, and increased protein oxidation [17]. Progerin is the main generator of mitochondrial dysfunction, as overexpression of exogenous progerin in 3T3L1 pre-adipocyte resulted in perturbed mitochondrial respiration, overproduction of superoxide anion and elevated ROS [18]. Likewise, the overexpression of progerin in rat nucleus pulposus (NP) cells caused alterations in mitochondrial morphology and dynamics, ATP generation and ΔΨ_m_ [49]. Thus, owing to the functional connection between defective mitochondria and HGPS pathogenesis, different strategies to recover mitochondrial function have been approached. Treatment of HGPS fibroblasts with the Ataxia Telangiectasia Mutated (ATM) inhibitor termed KU-60019 improved ΔΨ_m_ and prevented ROS accumulation, which was ultimately reflected in a reduction of senescent cells [50]. The authors claimed that such a therapeutic effect is based on the metabolic reprograming of HGPS cells, from glycolysis to oxidative phosphorylation, and the concomitant KU-60019-mediated clearance of progerin. Likewise, the exposure of HGPS fibroblasts to the antioxidant compound, methylene blue, enhanced different parameters of mitochondrial function, including mitochondrial morphology and mobility, ΔΨ_m_, and decreased ROS levels, which in turn resulted in a delay of cellular senescence. Methylene blue mainly acts by upregulating PGC-1α expression, a master transcription cofactor of mitochondrial biogenesis [20]. On the other hand, the incubation of HGPS fibroblasts with the Rho-associated coiled-coil kinase 1 (ROCK1) inhibitor named Y-27632, normalized mitochondrial respiration and reduced ROS levels by shifting the metabolic program of HGPS fibroblasts from glycolysis to oxidative phosphorylation [16]. Finally, treatment with sulforaphane, an activator of the transcription factor NRF2, a master regulator of the antioxidant defense [51], enabled progerin-expressing NP cells to recover mitochondrial function, namely improved ΔΨ_m_, increased ATP levels, and reduced ROS levels [49].

In this context, we previously reported that treatment of HGPS fibroblasts with LMB, a specific inhibitor of exportin CRM1, alleviated a series of senescent marks, including the aberrant nuclear morphology, expanded nucleoli, senescent cell morphology, and loss of peripheral heterochromatin and Lamin B1 downregulation [9]. To expand the evaluation of LMB as a potential therapeutic drug for HGPS, we ascertained in this study whether LMB has a beneficial effect on mitochondrial function. Remarkably, we found that treatment of HGPS cells with LMB elicits a recovery of mitochondrial function. The aberrant mitochondrial morphology characterized by reduced branching with fragmented/swollen mitochondria was shifted to a reticular network upon exposure to LMB. Consistent with a restored morphology, mitochondria from LMB-treated HGPS cells showed improved ΔΨ_m_, increased ATP content, and reduced ROS levels compared with vehicle-treated HGPS cells.

Mitochondrial function is governed by two antagonistic pathways: mitochondrial biogenesis and mitophagy, which work in a tight coordination to maintain cell homeostasis [52,53]. While newly synthetized mitochondria are generated by mitochondrial biogenesis, mitophagy conducts the elimination of defective mitochondria. Therefore, we envisaged that the effect of LMB on mitochondrial function might be based on the modulation of these processes. We evaluated PGC-1α expression as an indicator of mitochondrial biogenesis because this transcription coactivator is a pivotal activator of mitochondrial genes involved in mitochondrial biogenesis [31,54]. Remarkably, treatment of HGPS cells with LMB partially retrieved the expression of PGC-1α at both mRNA and protein levels, which occurred concomitantly with an increase in mitochondrial mass. The pharmacological inhibition of exportin CRM1 is intended to normalize the aberrantly enhanced nuclear protein export pathway found in HGPS cells [9]. Thus, we speculated that the attenuation of exportin CRM1 activity could favor the accumulation of transcription factors regulated by CRM1 that might activate the PGC-1α promoter such as the Nuclear Factor of Activated T cells (NFAT) and TFEB [36,37,55,56]. Sustaining this idea, TFEB mislocalization from the nucleus to the cytoplasm that occurs in HGPS was prevented by LMB treatment, which in turn probably contributes to enhance PGC-1α expression. Intriguingly, WT cells treated with LMB showed decreased PGC-1α expression. We hypothesized that Sirtuin 1 (Sirt1) a CRM1 client that has been involved in PGC-1α repression [57], accumulates in the nuclei of LMB-treated WT cells, driving to PGC-1α transcriptional repression. Owing to the fact that progerin activates Sirt1 [58], such an upregulation may then account for the repression of PGC-1α levels in HGPS cells, and for the rescue of expression upon treatment with LMB. Further experiments are required to prove this hypothesis. The recovery of PGC-1α expression could also be implicated in the reduction of ROS levels observed in LMB-treated HGPS cells, because it is a transcriptional coactivator of genes encoding ROS-detoxifying enzymes [54]. Intriguingly, the treatment of HGPS cells with LMB alleviated cytoplasmatic but not mitochondrial ROS. According to reference [59], we suggest that such a differential effect might be due to an unequal subcellular distribution of the redox system components in HGPS cells. It is clear that future investigation is required to solve this enigma.

On the other hand, we found a basal activation of mitophagy in HGPS cells, as demonstrated by the increase of both LC3-II/LC3-I ratio and PINK1 and parkin levels, as well as the increased colocalization of damaged mitochondria with LC3. Despite that, defective mitochondria with impaired ΔΨ_m_ accumulates in HGPS cells, which implies that mitophagy works inefficiently in HGPS cells. Since a reduction in the lysosomal content, decreased LAMP2 levels and the unexpected lack of colocalization between swollen mitochondria and lysosomes were found in HGPS cells, we speculate that the last steps of mitophagy, namely the fusion between autophagosomes containing defective mitochondria and lysosomes, is compromised in HGPS cells. Specifically, it has been reported that the lack of LAMP2 leads to defective lysosomal-dependent cargo degradation, which in turn provokes the accumulation of autophagosomes [43,60]. Consistently with our results, the accumulation of defective mitochondria caused by impaired mitophagy was previously observed in the progeroid syndromes Xeroderma pigmentosum and Cockayne [61].

Remarkably, all of the indicators of mitophagy were reversed in response to the treatment of HGPS cells with LMB, including the LC3-II/LC3-I ratio, PINK1 and Parkin levels and, moreover, the colocalization of damaged mitochondria with LC3. Concomitantly, LMB-treated HGPS cells exhibit a recovery of lysosomal content. The latter could be functionally linked to the nuclear accumulation of TFEB in HGPS cells treated with LMB (see above), because TFEB is a key transcription regulator for the expression of lysosomal and autophagy genes, which in turn support the biogenesis of lysosomes and autophagosomes, respectively [62,63]. Collectively, these results imply that impaired mitochondrial function leads to mitophagy activation in HGPS cells as an attempt to remove defective mitochondria. It is likely that mitochondrial abnormalities accumulate in HGPS cells as long as they remain in a non-proliferative but metabolically active stage. However, after three days of LMB treatment that resulted in the recovery of mitochondrial morphology/function, the activation of mitophagy is no longer required.

In summary, we demonstrated that the LMB-driven pharmacological inhibition of CRM1 rescues the mitochondrial function of HGPS cells via an improvement of mitochondrial biogenesis and lysosomal content. Furthermore, we analyzed, what we believe to be for the first time, the mitophagy pathway in HGPS cells. The normalization of the nuclear export mechanism using specific inhibitors of CRM1 is a promising strategy to fight HGPS.

## Data Availability

Not applicable.

## Supplementary Materials

The following supporting information can be downloaded at: https://www.mdpi.com/article/10.3390/cells12020275/s1, Figure S1: Validation of JC-1-based mitochondrial membrane potential and mitochondrial superoxide (MitoSox) assays standardization. (A) WT cells, previously treated with 12 μM FCCP (mitochondrial membrane uncoupler), were stained with fluorophore JC-1 for 15 min, and then subjected to confocal microscope analysis. Typical images of JC-1-stained cells are shown. Scale bar, 20 μm. (B) WT and HGPS-1 cells were stained with MitoSox, to measure the production of mitochondrial superoxide by flow cytometry (10,000 events) using two different concentrations of MitoSox (1 μM and 2.5 μM). We choose 1 μM MitoSox for further experiments; Figure S2: Increased colocalization of LC3 puncta and mitochondria in HGPS-1 cells is normalized as WT cells in the presence of LMB. (A) WT and HGPS-1 cells grown on coverslips were treated for 3 days with 1 nM LMB or the vehicle alone. Cells were then stained with MTR to decorate mitochondria and then fixed and immunolabeled for LC3. Nuclei were stained with DAPI prior to confocal microscopy analysis. Typical orthogonal views of the images from three separate experiments are shown and arrows point out LC3 and mitochondria colocalization. Scale bar 10 μm. the yellow arrow indicates colocalization. (B) The number of LC3 puncta colocalizing with mitochondria was quantified and data is shown in box and whiskers graph (percentile: 10–90, from three independent experiments, *n* = 60 cells per condition), and significant differences were obtained using nonparametric Mann Whitney test; ** *p* < 0.01; **** *p* < 0.0001.
