# Supplementary material for: Rescue of Mitochondrial Function in Hutchinson-Gilford Progeria Syndrome by the Pharmacological Modulation of Exportin CRM1
Source: Cells. 2023 Jan 10;12(2):275. doi: 10.3390/cells12020275 (PMC9856861; doi:10.3390/cells12020275)
Supplement: Supplementary file 1 [file cells-12-00275-s001.zip › cells-2036734-supplementary.pdf]

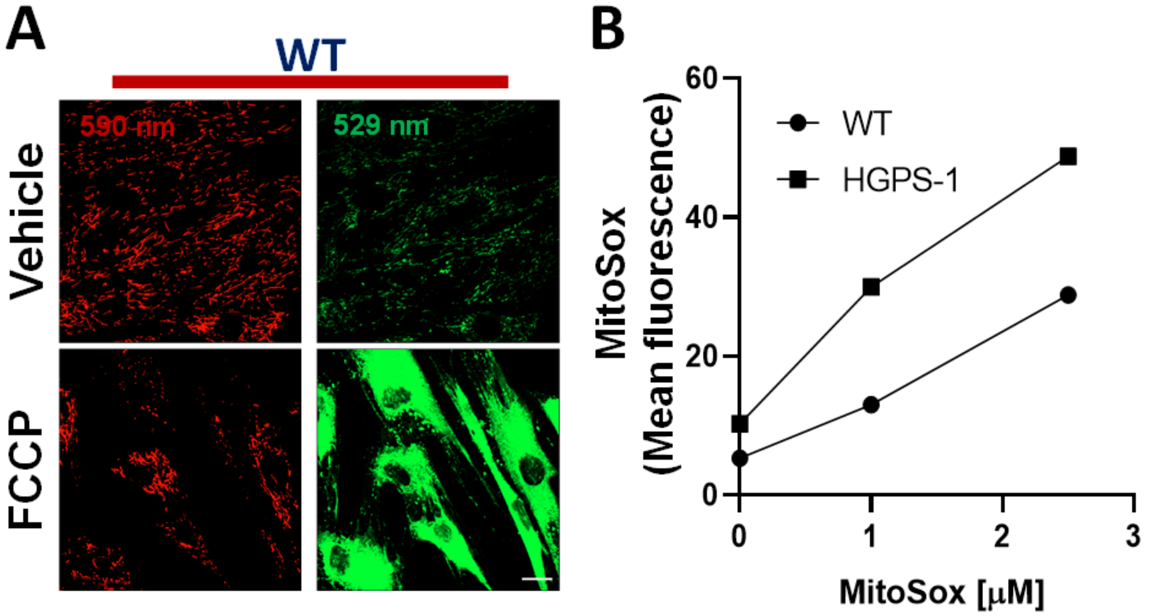

Supplementary figure S1. (A) Validation of JC-1-based mitochondrial membrane potential and mitochondrial superoxide (MitoSox) assays standardization. (A) WT cells, previously treated with 12 µM FCCP (mitochondrial membrane uncoupler), were stained with fluorophore JC-1 for 15 minutes, and then subjected to confocal microscope analysis. Typical images of JC-1-stained cells are shown. Scale bar, 20 µm. (B) WT and HGPS-1 cells were stained with MitoSox, to measure the production of mitochondrial superoxide by flow cytometry (10,000 events) using two different concentrations of MitoSox (1 µM and 2.5 µM). We choose 1 µM MitoSox for further experiments.

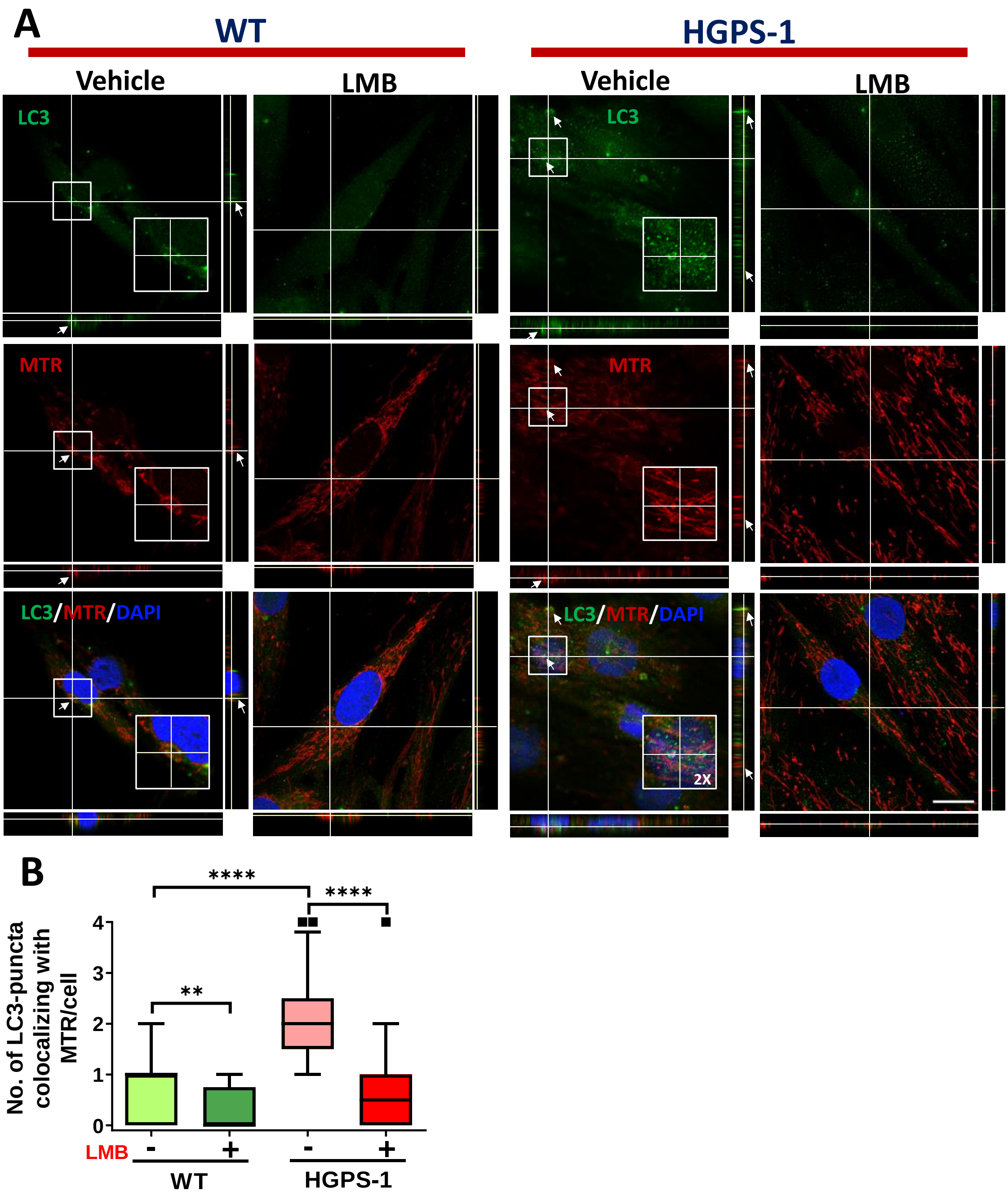

Supplementary figure S2. Increased colocalization of LC3 puncta and mitochondria in HGPS-1 cells is normalized as WT cells in the presence of LMB. (A) WT and HGPS-1 cells grown on coverslips were treated for 3 days with 1 nM LMB or the vehicle alone. Cells were then stained with MTR to decorate mitochondria and then fixed and immunolabeled for LC3. Nuclei were stained with DAPI prior to confocal microscopy analysis. Typical orthogonal views of the images from three separate experiments are shown and arrows point out LC3 and mitochondria colocalization. Scale bar 10 µm. the yellow arrow indicates colocalization. (B) The number of LC3 puncta colocalizing with mitochondria was quantified and data is shown in box and whiskers graph (percentile: 10-90, from three independent experiments, n= 60 cells per condition), and significant differences were obtained using nonparametric Mann Whitney test; $^{**}p < 0.01$; $^{****}p < 0.0001$.
